# From the Outside, Looking in: Photovoice as a Transformative Method in Community-Engaged Research

**DOI:** 10.1093/geroni/igaf122.1064

**Published:** 2025-12-31

**Authors:** Rachel Weldrick, Sheila Boamah, Samantha Biglieri, Atiya Mahmood, Sarah Canham

**Affiliations:** Concordia University, Montreal, Quebec, Canada; McMaster University, Hamilton, Ontario, Canada; Toronto Metropolitan University, Toronto, Ontario, Canada; Simon Fraser University, Vancouver, British Columbia, Canada; University of Utah, Salt Lake City, Utah, United States

## Abstract

Photovoice is a powerful tool in gerontological research and can bridge the gap between academia and community. Moreover, inviting participants and co-researchers to photograph their lived and embodied realities through photovoice offers researchers meaningful opportunities to learn from participants, while fostering transformative collaborations that challenge the status quo in social research. This paper explores the transformative aspects of photovoice methods in community-engaged research by synthesizing findings from three photovoice studies exploring diverse aging experiences. First, a photovoice study with older people living in temporary supportive housing illuminated the lived realities of housing precarity in later life and spurred arts-based community engagement and advocacy efforts. Second, a photovoice study with family caregivers to persons living in long-term care during COVID-19 lockdowns provided academic team members with glimpses into hidden experiences which would have been otherwise inaccessible. This process also served as a meaningful outlet for caregiver expression and connection during this time. Third, a case study in a suburban neighbourhood during COVID-19 included persons living with dementia as living experts of the embodied realities of dementia while problematizing exclusionary built environments. All three projects illuminate the transformative aspects of photographs in building partnerships and eliciting reflexivity. Findings exemplify the means through which academic and community partners can use photovoice to navigate the insider/outsider status in research and embed elements of advocacy into community-engaged research partnerships. As a critical and transformative method, photovoice can inform policy that is directly influenced by lived experiences to meaningfully address aging and health inequities.

